# Case of Ovarian Abscess, with Rupture into the Cavity of the Abdomen

**Published:** 1839-02-16

**Authors:** H. S. Patterson


*Case of Ovarian Abscess, with Rupture into the Cavity of the Abdomen.* By H. S. PATTERSON, M. D.

M. S., a negress, aged twenty, with procidentia uteri, came under my care as a dispensary patient in July last. She had never been pregnant, and menstruated regularly. She stated that her uterus was first prolapsed by a strain in lifting, about a year before. It was then "put up" by a physician; but pain in the back and sides, with profuse leucorrhœa and ardor urinæ, had been constant since. The procidentia was of several weeks' standing. The uterus was very tender to the touch,—and any attempt to replace it gave the sensation of raising a large mass, producing, at the same time, excruciating pain in the back and hypogastrium. Finding this, I desisted and ordered absolute rest, cups to the loins, laxative, and a saturnine lotion. Leaving town a few days after, I placed her under the care of my friend Dr. G. R. Winter, who, perceiving that the exposure of the uterus caused painful excoriation, succeeded in replacing it within the labia. This relieved the discharge and scalding, but she remained otherwise the same until the evening of August 14th, when she was suddenly attacked with intense pain in the abdomen, which increased rapidly, and early next morning she expired.

*Autopsy*, nine hours after death, assisted by Drs. Warrington and B. F. Hardy. The cavity of the abdomen was found to contain a very considerable quantity of thin, purulent matter. The peritoneum, in its lower portions, was much injected. The fallopian tubes and ovaries were of a dark purple colour, and greatly enlarged,—the latter to the size of an orange. The large intestines adhered to these in several points, and about them were numerous hydasids of various sizes. The appendicula vermiformis was firmly adherent to the right ovary, and at its extremity was an opening of the size of a quill, from which pus flowed on pressure. A probe inserted into this opening, passed freely upward into the cæcum, and downward into a cavity in the substance of the ovary. The mucous membrane of the appendicula presented several ulcerated spots, and could be traced distinctly to the opening. Nothing remained of the ovary but the thin walls of an abscess, the internal surface of which was rough, ragged, and of a dark purple. The pus contained had also a reddish tinge. The left ovary was in the same condition, but still entire. The uterus was normal.

The most interesting fact connected with this case, is the condition of the appendicula vermiformis. Had not the unfortunate rupture into the peritoneal cavity taken place, there can be no doubt that a way would have been provided, through which the contents of the abscess might be discharged into the bowel.

